# Association of neighborhood greenness with severity of hand, foot, and mouth disease

**DOI:** 10.1186/s12889-021-12444-7

**Published:** 2022-01-06

**Authors:** Zhicheng Du, Boyi Yang, Bin Jalaludin, Luke Knibbs, Shicheng Yu, Guanghui Dong, Yuantao Hao

**Affiliations:** 1 Department of Medical Statistics, School of Public Health, Sun Yat-sen University, Guangzhou, 510080 China; 2 Department of Occupational and Environmental Health, School of Public Health, Sun Yat-sen University, Guangzhou, 510080 China; 3 School of Public Health and Community Medicine, University of New South Wales, Kensington, NSW 1871 Australia; 4 School of Public Health, The University of Sydney, Camperdown, NSW 2006 Australia; 5 Chinese Center for Disease Control and Prevention, Beijing, 102206 China

**Keywords:** Hand, foot, and mouth disease, Neighborhood greenness, Effect modification, Population density

## Abstract

**Background:**

Hand, foot, and mouth disease (HFMD) is an epidemic infectious disease in China. Relationship of neighborhood greenness with human health has been widely studied, yet its association with severe HFMD has not yet been established.

**Methods:**

Individual HFMD cases that occurred in Guangdong province in 2010 were recruited and were categorised into mild and severe cases. Residential greenness was assessed using global land cover data. We used a case-control design (i.e., severe versus mild cases) with logistic regression models to assess the association between neighborhood greenness and HFMD severity. Effect modification was also examined.

**Results:**

A total of 131,606 cases were included, of whom 130,840 were mild cases and 766 were severe cases. In an unadjusted model, HFMD severity increased with higher proportion of neighborhood greenness (odds ratio, OR = 1.029, 95%CI: 1.009–1.050). The greenness-HFMD severity association remained (OR = 1.031, 95%CI: 1.006–1.057) after adjusting for population density, demographic variables and climate variables. Both population density (Z = 4.148, *P* < 0.001) and relative humidity (Z = -4.297, *P* < 0.001) modified the association between neighborhood greenness and HFMD severity. In the stratified analyses, a protective effect (OR = 0.769, 95%CI: 0.687–0.860) of greenness on HFMD severity were found in the subgroup of population density being lower than and equal to 5 ln(no.)/km^2^. While in both the subgroups of population density being higher than 5, the greenness had hazard effects (subgroup of > 5 & ≤7: OR = 1.071, 95%CI: 1.024–1.120; subgroup of > 7: OR = 1.065, 95%CI: 1.034–1.097) on HFMD severity. As to relative humidity, statistically significant association between greenness and HFMD severity was only observed in the subgroup of being lower than and equal to 76% (OR = 1.059, 95%CI: 1.023–1.096).

**Conclusions:**

Our study found that HFMD severity is associated with the neighborhood greenness in Guangdong, China. This study provides evidence on developing a prevention strategy of discouraging the high-risk groups from going to the crowded green spaces during the epidemic period.

**Supplementary Information:**

The online version contains supplementary material available at 10.1186/s12889-021-12444-7.

## Main finding

We found that neighborhood greenness was statistically associated with HFMD severity and that the association was modified by population density and relative humidity.

## Introduction

Greenness, defined as the earth’s surface consisting of green vegetation, has been greatly reduced worldwide due to urbanization. The relationships between neighborhood greenness and various human health outcomes have been widely studied [1], of which non-communicable diseases were usually investigated. A beneficial association has been found for greenness and non-communicable diseases including overweight and obesity [2, 3], mental health [4], birth and developmental outcomes [5], cardiovascular outcomes [6], and mortality [7]. In addition, higher greenness levels have also been reported to be significantly associated with lower odds of chronic conditions in older populations [8], with greater physical time as well as physical and social activity [9]. However, the associations between greenness and communicable diseases have been rarely studied. Increased neighborhood greenness may encourage people to spend more time outdoors [10], and thus more opportunities for contact transmission of communicable diseases. Thus, it is plausible that higher greenness may be associated with higher incidence of communicable diseases.

Hand, foot, and mouth disease (HFMD) has attracted the attention of the public in China. HFMD is a infectious disease mainly occurred in children under 5 years of age and spread primarily through close contact. In China, legally reported infectious diseases are classified into 3 categories according to the degree of danger: Category A, B and C. The annual incidence of HFMD in China was 168/100,000 population during the past 5 years (i.e., 2014–2018), accounting for 57% cases in the category C notifiable diseases [11]. Moreover, severe HFMD cases may lead to serious complications such as myocarditis, aseptic meningitis, and pulmonary edema and even death [12]. The infectious diseases surveillance systems often collect information only on positive cases rather than healthy individuals. Many surveillance system-based studies are conducted only on positive cases. For HFMD, the severity of the cases is of concern and many studies have investigated risk factors for HFMD severity [13]. The evidence suggests that, in addition to the clinical features [14], environmental factor may also play an important role in the development of severe HFMD [15]. In addition, many genotypes of enteroviruses can cause HFMD, and the severity caused by different genotypes of enteroviruses varies with EV71, CVA6, and CVA16 causing the most part of severe cases [16].

There may be a potential link between green space and HFMD severity. Enteroviruses (e.g., HFMD-related viruses) are most commonly associated with either mild or asymptomatic infections [17]. A relative high rate (8.11%) of asymptomatic infection of HFMD among children was previously revealed [18]. The multiple complex contact during their activities on the greenness may increase the probability of HFMD-related viruses infection, as well as reinfection or coinfection. Reinfection and coinfection of infectious diseases usually developed severe symptoms [19]. However, the association between greenness and HFMD severity have been rarely investigated.

We hypothesized that neighborhood greenness is associated with HFMD severity. We tested this hypothesis by linking HFMD cases from Guangdong province, southern China in 2010 to neighborhood greenness levels.

## Materials and methods

### Study area

**Fig. 1:**
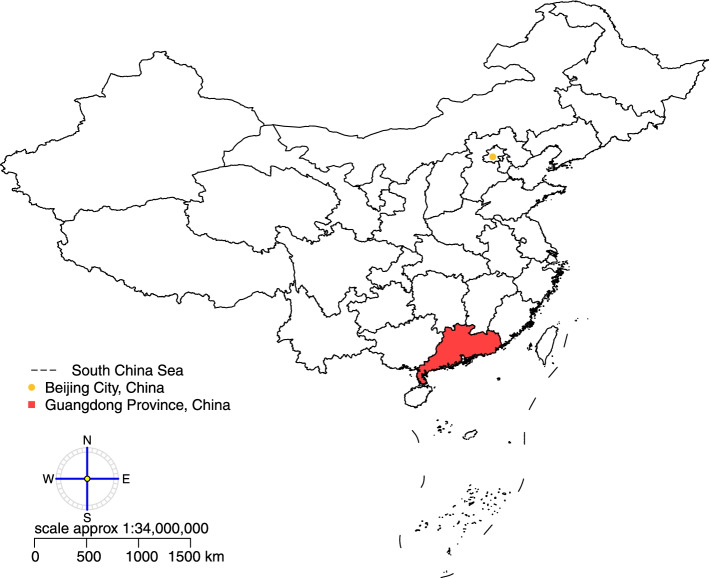
Location of Guangdong Province in China

Guangdong is a coastal province in South China and north of the South China Sea (Fig. 1). With a population of 115 million (as of 2019) across a total area of about 179,800 km^2^, Guangdong is the most populous province of China. Its economy is larger than that of any other province in the nation. Guangdong has a humid subtropical climate, though nearing a tropical climate in the far south. Winters are short, mild, and relatively dry, while summers are long, hot, and very wet.

### Outcome

A total of 226,619 HFMD cases in Guangdong province in 2010 were reported to the China Information System for Disease Control and Prevention. First, we excluded 80,625 cases because they didn’t have complete address information. For example, it might be that the patient refuses to provide address information, or that the address information is only specific to the village and cannot be accurately located. The demographic distribution of these excluded cases were similar to those of the included cases (Table S1). Second, we excluded 14,042 cases older than 5 years of age because HFMD is recognized as commonly occurring in children under 5 years of age [20]. Third, we excluded 346 cases registered with the wrong information (e.g., the “teacher” was assigned to a child). Finally, 131,606 cases with 766 severe cases diagnosed according to guidelines for diagnosis and treatment of HFMD [21] were included in this study. The diagnosis of HFMD cases can be made by combining epidemiological history, clinical presentation and etiological examination, including both clinically diagnosed cases and confirmed cases. HFMD cases with one of the following clinical manifestations were identified as severe cases: 1) persistent high fever; 2) neurological manifestations; 3) respiratory abnormalities; 4) circulatory dysfunction; 5) elevated peripheral blood leukocyte count; 6) elevated blood glucose; and 7) elevated blood lactate. In addition to the residential address and children’s age (birth date and onset date), other information including sex and whether home-care was delivered (which means the children are taken care at home rather than nursery school) were also collected by the China Center for Disease Control (CDC) under a legal mandate. The onset date was transformed to onset season as: Spring (March–May), Summer (June–August), Autumn (September–November) and Winter (December–February). We geocoded the residential address specific to buildings using the Geocoder service of Baidu Maps [22].

### Greenness

**Fig. 2:**
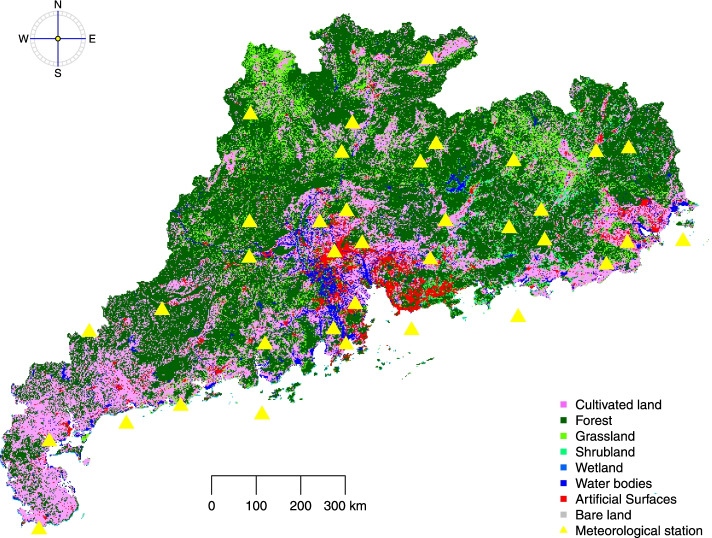
Land cover and meteorological stations of Guangdong Province

Neighborhood greenness levels were extracted from the global land cover data (GlobeLand30, http://www.globallandcover.com/GLC30Download/index.aspx), with a resolution of 30 m × 30 m. There were ten types of land cover are recorded in GlobelLand30, and eight types were found in Guangdong Province (Fig. 2). In this study, we classified five types of land cover (cultivated land, forest, grassland, shrubland, and wetland) as greenness. The NDVI levels of different types of land cover were extracted (Table S8). We calculated the neighborhood greenness as the proportion of the greenness within 500 or 1000 m circular buffer of each residential address. To be consistent with previous studies [23], we used 500 m buffers in the main analysis, and present the results for 1000 m buffers in the supplementary materials (Table S2, S3, S4, S5, and Fig. S1).

### Covariates

We collected daily average air temperature and relative humidity from 36 weather stations in Guangdong province from the China Meteorological Data Service Center (CMDC, http://data.cma.cn/en). Each HFMD case was assigned the annual average air temperature and relative humidity using the inverse distance weighted (IDW) interpolation method [24].

We downloaded 1 × 1 km^2^ population density map (Gridded Population of the World, GPW) from NASA Socioeconomic Data and Applications Center (SEDAC, https://sedac.ciesin.columbia.edu/) for Guangdong province. Each HFMD case was assigned population density at their residential location by extracting the information directly from the population density map. Since the distribution of population density was positively skewed, the natural log transformation (i.e., ln) was taken for population density data before statistical analysis.

### Statistical analyses

First, we used descriptive analyses to assess the distribution of greenness, demographic, climate factors, and population density stratified by the HFMD severity. Second, we used the smoothed scatter plots between the logit values and the independent variables to check the linearity assumption of the logistic regression (Fig. S2). Except for the relative humidity, the other variables are largely consistent with the assumption of linearity. Thus, we recoded the relative humidity into binary variable using the cutoff value of 76% according to the location of the knot in the smoothed scatter plot. Third, we used logistic regression models to investigate unadjusted associations between neighborhood greenness and HFMD severity. Fourth, we estimated the association after adjusting for population density, demographic variables (age, sex, and home-care or not), and climate variables (temperature, relative humidity, and season of onset). Fifth, we explored effect modification by population density, age, sex, home-care, temperature, relative humidity and onset season by including interaction terms in the logistic regression models. Sixth, we used line plots with 95%CI (confidence interval) to visualize the interaction effects for the continuous variables. The cutoff values were chosen according to the overlap between the confidence band and the null effect line. Finally, we conducted stratified logistic regression models to quantify the odds ratio (OR) of the neighborhood greenness on HFMD severity. Except for when there were statistically significant interactions, the stratified analyses results for all the other covariates are reported in the supplementary materials (Table S6). In addition, we used the increase of 10% instead of 1% to calculate the ORs and 95%CIs for the neighborhood greenness avoiding the small coefficients. R version 4.0.2 (R Foundation for Statistical Computing, Vienna, Austria; https://www.r-project.org/) was used for all data management and statistical analyses.

## Results

Table 1 shows the descriptive statistics of greenness, demographic, climate factors, and population density by HFMD severity. The proportion of greenness within 500 m buffers for mild and severe cases were 28.7 and 32.2%, respectively (*P* = 0.005). Severe cases were slightly younger than the mild cases (2.1 vs. 2.4 years of age, *P* < 0.001). The severe cases had more home-care children than the mild cases (83.8% vs. 76.1%, *P* < 0.001). Average air temperature among the severe cases was a bit higher than that of the mild cases (22.2 vs. 22.1 °C, *P* < 0.001). Population density among the severe cases was only slightly lower than that of the mild cases (7.8 vs. 7.9 ln(no.)/km^2^, *P* = 0.049).

**Table 1:** Descriptive statistics of exposure, demographic, and environment variables by HFMD severity

Variable	Mild cases ***n*** = 130,840	Severe cases ***n*** = 766	***P*** value
Exposure variable, *Mean* ± *SD*			
Greenness (500 m)	28.7 ± 34.0	32.2 ± 33.2	0.005
Greenness (1000 m)	33.9 ± 31.9	37.2 ± 30.3	0.004
Demographic variables			
Age (year), *Mean* ± *SD*	2.4 ± 1.2	2.1 ± 1.1	< 0.001
Sex, *n*(%)			0.226
Male	84,347 (64.5)	510 (66.6)	
Female	46,493 (35.5)	256 (33.4)	
Home-care, *n*(%)			< 0.001
Yes	99,609 (76.1)	642 (83.8)	
No	31,231 (23.9)	124 (16.2)	
Population density (ln(no.)/km^2^), *Mean* ± *SD*	7.9 ± 1.5	7.8 ± 1.3	0.049
Environment variables, *Mean* ± *SD*			
Temperature (°C)	22.1 ± 0.5	22.2 ± 0.4	< 0.001
Relative humidity (%)	75.9 ± 1.7	75.9 ± 1.6	0.865
Relative humidity			0.023
< 76	76,131 (58.19)	477 (62.27)	
≥ 76	54,709 (41.81)	289 (37.73)	
Onset season, *n*(%)			< 0.001
Spring	50,771 (38.80)	330 (43.08)	
Summer	43,521 (33.26)	306 (39.95)	
Autumn	26,590 (20.32)	98 (12.79)	
Winter	9958 (7.61)	32 (4.18)	

Higher neighborhood greenness levels were statistically associated with HFMD severity in the crude model (OR = 1.029, 95%CI: 1.009–1.050, *P* = 0.005) (Table 2). This association remained significant (OR = 1.031, 95%CI: 1.006–1.057, *P* = 0.015) after adjusting for population density, demographic variables (age, sex, and home-care) and environment variables (temperature, relative humidity, and onset season). The univariate models were also conducted to explore the crude relationships between HFMD severity and the covariates (Table S7).

**Table 2:** Logistic regression models between the HFMD severity and the neighborhood greenness

Greenness(500 m)	***OR***	95%***CI***	***P*** value
Crude model	1.029	(1.009, 1.050)	0.005
Adjusted model^a^	1.031	(1.006, 1.057)	0.015

*OR* Odds ratio, *CI* Confidence interval

^a^Adjusted for population density, demographic variables (age, sex, home-care) and environment variables (temperature, relative humidity, onset season)

Statistically significant interaction effects were observed for both population density (Z = 4.148, *P* < 0.001) and relative humidity (Z = -4.297, *P* < 0.001) (Table 3). Age, sex, home-care, temperature, and onset season did not modify the associations between neighborhood greenness and HFMD severity.

**Table 3:** Logistic regression models with interaction term with neighborhood greenness on HFMD severity

Interaction term^a^	*Z* value	***P*** value
Greenness(500 m) × population density	4.148	< 0.001
Greenness(500 m) × age	0.305	0.760
Greenness(500 m) × sex	−1.472	0.141
Greenness(500 m) × home-care	−0.350	0.726
Greenness(500 m) × temperature	−0.722	0.471
Greenness(500 m) × relative humidity	−4.297	< 0.001
Greenness(500 m) × onset season	−0.363	0.717

^a^All models were adjusted for population density, demographic variables (age, sex, home-care) and environment variables (temperature, relative humidity, onset season)

**Fig. 3:**
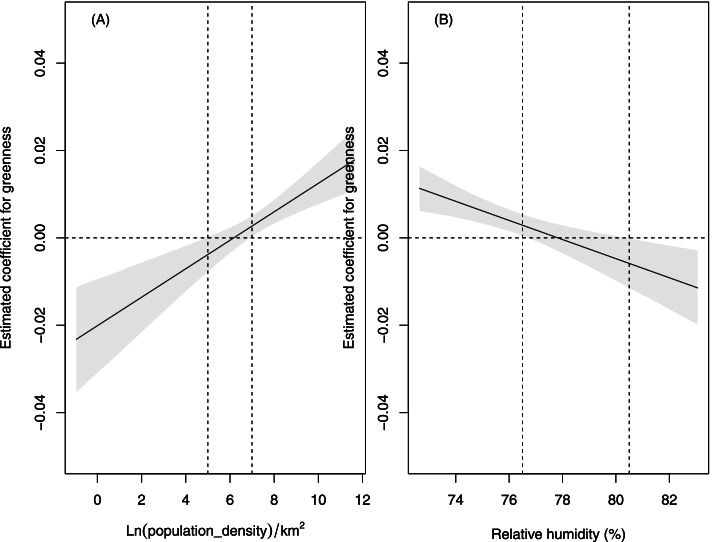
Interaction effects for the neighborhood greenness (500 m) by different levels of population density

Figure 3 presents the estimated coefficients for the neighborhood greenness by levels of population density and relative humidity. The estimated coefficients were derived from the results of the logistic regression models in Table 3. With increasing levels of population density, the coefficients for greenness increased from negative to positive. When the levels of population density were lower than or equal to 5 ln(no.)/km^2^, there was a protective effect of greenness on HFMD severity, while there was a hazard effect when the levels of population density were higher than 7 ln(no.)/km^2^.

Table 4 shows the relationship between the HFMD severity and the neighborhood greenness stratified by the population density using logistic regression models. When population density was lower or and equal to 5 ln(no.)/km^2^, greenness had a protective effect (OR = 0.769, 95%CI: 0.687–0.860) on HFMD severity. When population density was greater than 5 ln(no.)/km^2^, greenness increased the risk for severe HFMD (> 5 ln(no.)/km^2^ & ≤7 ln(no.)/km^2^: OR = 1.071, 95%CI: 1.024–1.120; > 7 ln(no.)/km^2^: OR = 1.065, 95%CI: 1.034–1.097). As to relative humidity, statistically significant association between greenness and HFMD severity was only observed when relative humidity was lower than or equal to 76% (OR = 1.059, 95%CI: 1.023–1.096).

**Table 4:** Logistic regression models between the HFMD severity and the neighborhood greenness stratified by the population density and relative humidity

Greenness (500 m)	***OR***	95%***CI***	***P*** value
Population density (ln(no.)/km^2^)			
≤ 5	0.769	(0.687, 0.860)	< 0.001
> 5 & ≤7	1.071	(1.024, 1.120)	0.003
> 7	1.065	(1.034, 1.097)	< 0.001
Relative humidity (%)			
< 76	1.059	(1.023, 1.096)	0.001
≥ 76	1.010	(0.975, 1.046)	0.587

All models were adjusted for population density, demographic variables (age, sex, home-care) and environment variables (temperature, relative humidity, onset season) except the stratified variable

Several sensitivity analyses were also conducted. First, the neighborhood greenness levels were recoded as binary variable by the median value. The OR for the crude model was 1.337 (95%CI: 1.158–1.543, *P* < 0.001), and 1.342 (1.145–1.574, *P* < 0.001) for the adjusted model. Second, the cases older than 5 years of age were included in the main models. The OR for the crude model was 1.029 (95%CI: 1.009–1.050, *P* = 0.004), and 1.031 (1.006–1.057, *P* = 0.015) for the adjusted model. Third, the hospitals were included as the random effect (Chi-square = 1892.8, *P* < 0.001) and estimated using generalized linear mixed model. The OR for the crude model was 1.093 (95%CI: 1.068–1.118, *P* < 0.001), and 1.034 (1.007–1.062, *P* = 0.014) for the adjusted model.

## Discussions

In this case-control study with a large sample size, we found that neighborhood greenness was statistically associated with HFMD severity and that the association was modified by population density and relative humidity. The greenness-HFMD risk increased when the population density was greater than 5 ln(no.)/km^2^ and the relative humidity was lower than or equal to 76%.

There are few publications in the scientific literature on greenness and HFMD severity, thus we are unable to compare our findings with other studies. However, there are a few studies that have explored greenness exposure and HFMD incidence [25, 26]. For example, one of our previous study found that NDVI (normalized difference in vegetation index), one of the metrics of greenness, was negatively associated with county-level HFMD incidence (Relative risk: 0.889, 95%CI: 0.883–0.895) [26]. In Shandong Province, NDVI was an important predictor of HFMD incidence at the district level [25]. These previous studies conducted the analysis using regional level data leading to exposure measurement bias [27], on which the study population in the same region were assigned the same level of exposure. Our study assigned greenness levels at the individual-level, which would have reduced exposure misclassification.

We found that effects of neighborhood greenness were dependent on population density. While a positive association of neighborhood greenness with HFMD severity was observed in children living in areas with higher population density, a negative association was observed in children living in areas with lower population density. A plausible explanation may be that the crowds and social gatherings can facilitate disease transmission [28] and cross-infection by multiple viruses. For example, densely populated areas are reported to be ideal for the development and spread of some respiratory epidemics [29], one of the transmission routes for HFMD [20]. Physical contacts, resulting in transmission of communicable diseases, would increase non-linearly with increasing population density in locations with increased neighborhood greenness. As to the negative association with lower population density, The mechanisms for the positive effects of greenness on human health have been previously reported [1]. For the protective effects on HFMD, the potential reasons could be: providing the locations for both routine and recreational physical activity to promote health [30], buffering exposure to air pollution (e.g., removing air pollutants) so as adversely affect the immune system [31], and alleviating thermal discomfort during heat stress [32].

The relationship between the population size/density and incidence of infectious diseases were revealed as inconsistent among different diseases (e.g., positively correlated with AIDS, rubella, measles, and syphilis; negatively with scrub typhus, legionellosis, and EHEC) [33]. The contact rate is one of the modifying factors of relationship between population density and infectious diseases, which is usually nonlinear [34]. For HFMD, in general, the higher the population density, the higher the contact rate, and the higher HFMD incidence. For example, the average population density of HFMD cases were higher than the average level of the whole province (mild cases: 7.9 and severe cases: 7.5 vs. 6.4 ln(no.)/km^2^) [35]. As for the neighborhood greenness of concern in this study, it provides a platform for translating population density into effective contact rates.

We also find that the effects of neighborhood greenness were modified by relative humidity. A statistical positive association of neighborhood greenness with HFMD severity was only observed in a subgroup living in areas with lower relative humidity. A plausible explanation may be that relative wet weather was more likely to correspond to the cultivated land (Pearson correlation coefficient, *r* = 0.238, *P* < 0.001) rather than that of grassland (*r* = − 0.017, *P* < 0.001). In addition, the cultivated land would discourage the outdoor activities of children than other types of greenness. Consistent with the potential mechanism of population density impacting on neighborhood greenness, the crowds and social gatherings in a relative dry weather can facilitate disease transmission and cross-infection by multiple viruses.

While our study has many strengths, including the large sample size and the design for analyzing the environmental factors at individual level to reduce the exposure misclassification, there were also several important limitations. First, there may be selection bias as we excluded many cases due to their incomplete information on residential address. These cases were more likely to be from the sub-urban region where covered with more greenness and the address details are difficult to state. The proportion of severe cases in these cases were higher than those included in the study (0.79% vs. 0.58%). This might result in an underestimate for the effects of neighborhood greenness on the HFMD severity. Second, only a limited number of covariates were included in our analyses due to the data availability. However, the covariates we used included both individual characteristics (e.g., age, sex, and home-care) and the known climate factors (e.g., air temperature and relative humidity). Third, the levels of the environmental factors were assigned with the annual average rather than specific for the day of onset. However, the climate of the Guangdong Province is subtropical to tropical with a mild climate and abundant rainfall and green all the year round. Thus, the environmental factors we used were relative stable across the seasons. Fourth, the greenness metric in our study takes into account cultivated land, forest, grassland, shrubland, and wetland rather than park and playing fields only. The fact may be that cases are coming from more crowed green space and the current results might be underestimated in the effects of greenness on HFMD severity. The government should build more green spaces suitable for residents’ activities. Our findings could be generalizable to other settings with similar socio-economic and environmental conditions. Fifth, there were updates in the typing criteria for HFMD cases over time [21, 36, 37]. The results might vary from year to year, and comparison of results between years requires caution. Sixth, as for any common, self-limiting illness, most HFMD cases go undetected because their condition is asymptomatic, or the patient does not seek formal care, or he/she is not diagnosed and reported [38]. Seventh, we cannot yet estimate the extent to which people use these land covers, so we assume that the cases all have the same access to the green spaces.

## Conclusions

Our findings suggest that the HFMD severity is associated with the neighborhood greenness levels in Guangdong, China. Moreover, the association was significantly modified by population density with positive associations in higher population density areas, and negative associations in lower population density areas. This study provides evidence for developing a prevention strategy of discouraging the high-risk groups from going to the crowded green spaces during the epidemic period to reduce the probability of case severity.

## Supplementary Information

**Additional file 1.**

## Data Availability

All the environmental factors including neighborhood greenness, air temperature, relative humidity, and population density we used are available as the open sources and can be acquired according to the provenance listed in the “Materials and methods” section. The HFMD cases data are available from China CDC (http://www.chinacdc.cn/en/), which were used under license and not publicly available.
