# Supplementary material for: Association of neighborhood greenness with severity of hand, foot, and mouth disease
Source: BMC Public Health. 2022 Jan 6;22:38. doi: 10.1186/s12889-021-12444-7 (PMC8739664; doi:10.1186/s12889-021-12444-7)
Supplement: Supplementary file 1 — Additional file 1. [file 12889_2021_12444_MOESM1_ESM.docx]

**Supplementary materials**

Table S1. Descriptive statistics of demographic variables by HFMD severity among the excluded cases due to the incomplete address information.

| **Variable** | **Mild cases**  ***n*=79987** | **Severe cases**  ***n*=638** | ***P* value** |
| --- | --- | --- | --- |
| Demographic variables |  |  |  |
| Age (year), *Mean*±*SD* | 2.7±2.4 | 2.2±1.6 | <0.001 |
| Sex, *n*(%) |  |  | 0.095 |
| Male | 52071 (65.1) | 436 (68.3) |  |
| Female | 27916 (34.9) | 202 (31.7) |  |
| Home-care, *n*(%) |  |  | 0.007 |
| Yes | 63111 (78.9) | 531 (83.2) |  |
| No | 16876 (21.1) | 107 (16.8) |  |
| Onset season, *n*(%) |  |  | <0.001 |
| Spring | 29800 (37.26) | 296 (46.39) |  |
| Summer | 28017 (35.03) | 268 (42.01) |  |
| Autumn | 15713 (19.64) | 53 (8.31) |  |
| Winter | 6457 (8.07) | 21 (3.29) |  |

Table S2. Logistic regression models between the HFMD severity and the neighborhood greenness

| **Greenness(1000m)** | ***OR*** | **95%*CI*** | ***P* value** |
| --- | --- | --- | --- |
| Crude model | 1.032 | ( 1.010 , 1.054 ) | 0.004 |
| Adjusted model* | 1.040 | ( 1.011 , 1.070 ) | 0.006 |

*OR*: Odds ratio; *CI*: Confidence interval.

*Model adjusted for population density, demographic variables (age, sex, home-care) and environment variables (temperature, relative humidity, onset season).

Table S3. Logistic regression models with interaction term with neighborhood greenness on HFMD severity

| Interaction term* | *Z* value | ***P* value** |
| --- | --- | --- |
| Greenness(1000m)×population density | 4.325 | <0.001 |
| Greenness(1000m)×age | -0.366 | 0.714 |
| Greenness(1000m)×sex | -1.346 | 0.178 |
| Greenness(1000m)×home-care | -0.331 | 0.740 |
| Greenness(1000m)×temperature | -0.678 | 0.498 |
| Greenness(1000m)×relative humidity | -4.816 | <0.001 |
| Greenness(1000m)×onset season | -0.516 | 0.606 |

*All models were adjusted for population density, demographic variables (age, sex, home-care) and environment variables (temperature, relative humidity, onset season).

Table S4. Logistic regression models between the HFMD severity and the neighborhood greenness stratified by the population density and relative humidty

| **Greenness(1000m)** | ***OR*** | **95%*CI*** | ***P* value** |
| --- | --- | --- | --- |
| Population density (ln(no.)/km^2^) |  |  |  |
| ≤5 | 0.734 | ( 0.645 , 0.835 ) | <0.001 |
| >5 & ≤7 | 1.083 | ( 1.028 , 1.141 ) | 0.003 |
| >7 | 1.083 | ( 1.048 , 1.119 ) | <0.001 |
| Relative humidity (%) |  |  |  |
| <76 | 1.080 | ( 1.037 , 1.125 ) | <0.001 |
| ≥76 | 1.011 | ( 0.972 , 1.052 ) | 0.570 |

*All models were adjusted for population density, demographic variables (age, sex, home-care) and environment variables (temperature, relative humidity, onset season) except the stratified variable.

Table S5. Logistic regression models between the HFMD severity and the neighborhood greenness stratified by the variables rather population density and relative humidty in the main text

| **Greenness(1000m)** | ***OR*** | **95%*CI*** | ***P* value** |
| --- | --- | --- | --- |
| Age (y) |  |  |  |
| ≤2 | 1.047 | ( 1.014 , 1.080 ) | 0.005 |
| >2 | 1.078 | ( 1.040 , 1.117 ) | <0.001 |
| Sex |  |  |  |
| male | 1.052 | ( 1.017 , 1.088 ) | 0.004 |
| female | 1.016 | ( 0.967 , 1.067 ) | 0.530 |
| Home-care |  |  |  |
| no | 1.009 | ( 0.936 , 1.089 ) | 0.809 |
| yes | 1.045 | ( 1.014 , 1.077 ) | 0.005 |
| Season |  |  |  |
| Spring | 1.058 | ( 1.012 , 1.106 ) | 0.013 |
| Summer | 1.041 | ( 0.997 , 1.088 ) | 0.068 |
| Autumn | 1.002 | ( 0.930 , 1.079 ) | 0.964 |
| Winter | 1.013 | ( 0.887 , 1.157 ) | 0.847 |
| Temperature (℃) |  |  |  |
| ≤22.24 | 1.068 | ( 1.023 , 1.115 ) | 0.003 |
| >22.24 | 1.027 | ( 0.990 , 1.066 ) | 0.153 |

*All models were adjusted for population density, demographic variables (age, sex, home-care) and environment variables (temperature, relative humidity, onset season) except the stratified variable. The medians of age and temperature were 2 (y) and 22.24 (℃).

Table S6. Logistic regression models between the HFMD severity and the neighborhood greenness stratified by the variables rather population density and relative humidty in the main text

| **Greenness(500m)** | ***OR*** | **95%*CI*** | ***P* value** |
| --- | --- | --- | --- |
| Age (y) |  |  |  |
| ≤2 | 1.034 | ( 1.004 , 1.064 ) | 0.024 |
| >2 | 1.071 | ( 1.037 , 1.106 ) | <0.001 |
| Sex |  |  |  |
| male | 1.042 | ( 1.012 , 1.074 ) | 0.006 |
| female | 1.007 | ( 0.964 , 1.052 ) | 0.748 |
| Home-care |  |  |  |
| no | 1.008 | ( 0.944 , 1.077 ) | 0.804 |
| yes | 1.035 | ( 1.007 , 1.063 ) | 0.013 |
| Season |  |  |  |
| Spring | 1.04 | ( 1.001 , 1.082 ) | 0.046 |
| Summer | 1.034 | ( 0.995 , 1.075 ) | 0.087 |
| Autumn | 1.009 | ( 0.944 , 1.079 ) | 0.782 |
| Winter | 0.998 | ( 0.889 , 1.121 ) | 0.973 |
| Temperature (℃) |  |  |  |
| ≤22.24 | 1.051 | ( 1.014 , 1.09 ) | 0.007 |
| >22.24 | 1.023 | ( 0.989 , 1.058 ) | 0.184 |

*All models were adjusted for population density, demographic variables (age, sex, home-care) and environment variables (temperature, relative humidity, onset season) except the stratified variable. The medians of age and temperature were 2 (y) and 22.24 (℃).

Table S7. Univariate Logistic regression models between the HFMD severity and the covarates.

|  | ***OR*** | **95%*CI*** | ***P* value** |
| --- | --- | --- | --- |
| Population density | 0.955 | ( 0.911 , 1.000 ) | 0.049 |
| Age (y) | 0.844 | ( 0.792 , 0.900 ) | <0.001 |
| Sex (ref.=Male) | 0.911 | ( 0.783 , 1.059 ) | 0.223 |
| Occupation | 1.623 | ( 1.339 , 1.968 ) | <0.001 |
| Temperature (°C) | 1.763 | ( 1.495 , 2.080 ) | <0.001 |
| Relative humitidy (%, ref.<76) | 0.843 | ( 0.728 , 0.976 ) | 0.022 |
| Onset season (ref.=Spring) |  |  |  |
| Summer | 1.082 | ( 0.925 , 1.264 ) | 0.324 |
| Autumn | 0.567 | ( 0.452 , 0.711 ) | <0.001 |
| Winter | 0.494 | ( 0.344 , 0.711 ) | <0.001 |

Table S8. NDVI levels of different types of land cover.

| Land cover | NDVI | | | |
| --- | --- | --- | --- | --- |
|  | Jan | Apr | Jul | Oct |
| Cultivated land | 0.504±0.161 | 0.497±0.188 | 0.409±188 | 0.586±0.141 |
| Forest | 0.602±0.145 | 0.635±0.160 | 0.561±0.156 | 0.513±0.195 |
| Grassland | 0.517±0.171 | 0.561±0.185 | 0.495±0.183 | 0.505±0.170 |
| Shrubland | 0.584±0.164 | 0.580±0.187 | 0.464±0.191 | 0.511±0.191 |
| Wetland | 0.579±0.307 | 0.581±0.289 | 0.447±0.399 | 0.499±0.359 |





Figure S1. Interaction effects for the neighborhood greenness (1000m) by different levels of population density.


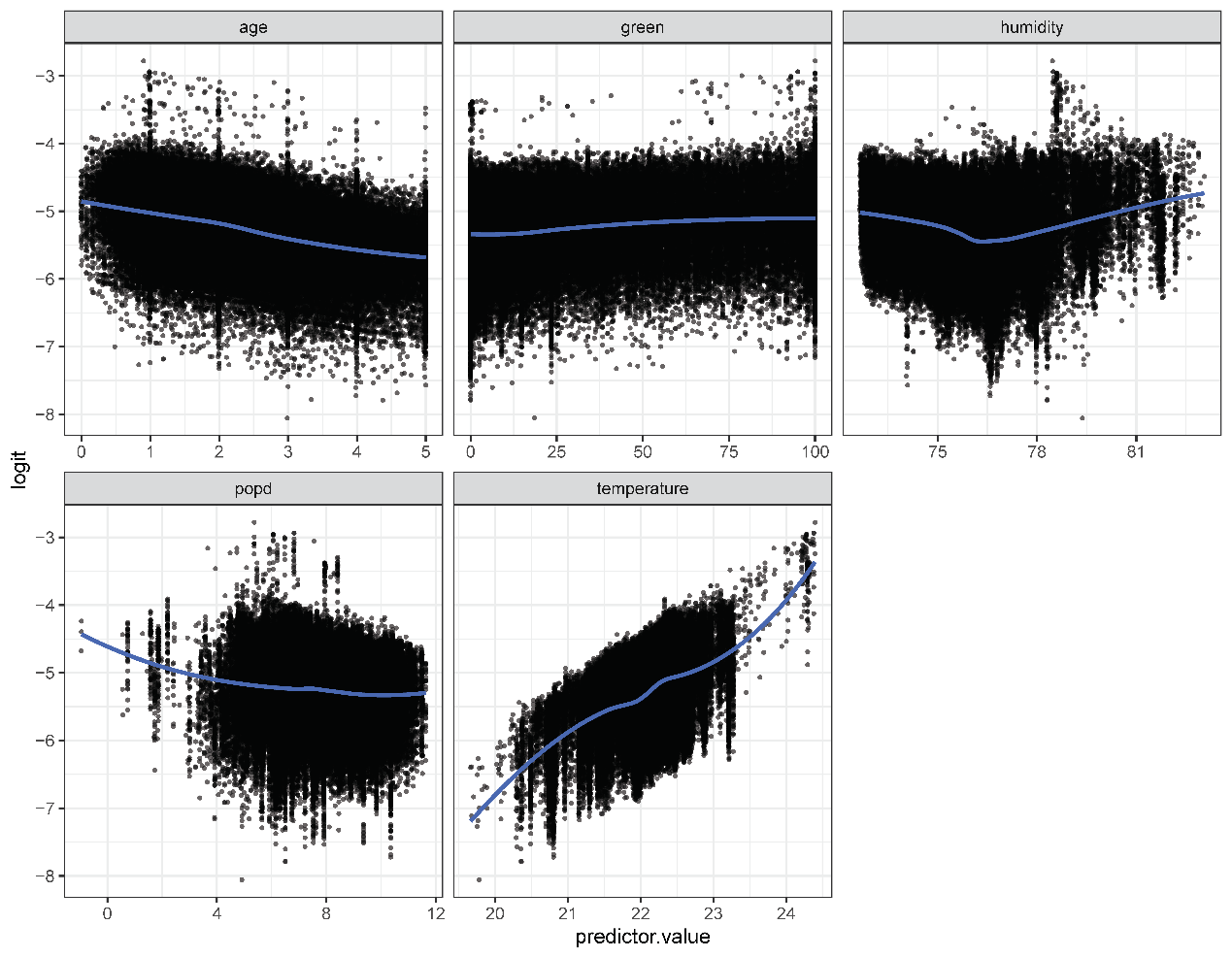


Figure S2. Smoothed scatter plots between the logit values and the independent variables.
